# Investigating the optical clearing effects of 50% glycerol in ex vivo human skin by harmonic generation microscopy

**DOI:** 10.1038/s41598-020-77889-z

**Published:** 2021-01-11

**Authors:** Jia-Hong Lai, En-Yu Liao, Yi-Hua Liao, Chi-Kuang Sun

**Affiliations:** 1 Department of Electrical Engineering and Graduate Institute of Photonics and Optoelectronics, National Taiwan University, Taipei, 10617 Taiwan; 2 Department of Dermatology, National Taiwan University Hospital and College of Medicine, National Taiwan University, Taipei, 10002 Taiwan

**Keywords:** Biophotonics, Imaging and sensing, Multiphoton microscopy, Tissues, Permeation and transport

## Abstract

Imaging depth and quality of optical microscopy can be enhanced by optical clearing. Here we investigate the optical clearing of the ex vivo human skin by 50% glycerol topical application, which is allowed for cosmetic usage. Harmonic generation microscopy, by combining second and third harmonic generation (THG) modalities, was utilized to examine the clearing effect. The THG image intensity is sensitive to the improved optical homogeneity after optical clearing, and the second harmonic generation (SHG) image intensity in the dermis could serve as a beacon to confirm the reduction of the scattering in the epidermis layer. As a result, our study supports the OC effect through 50% glycerol topical application. Our study further indicates the critical role of stratum corneum shrinkage for the observed SHG and THG signal recovery.

## Introduction

Optical microscopy is a light-based technique with a resolving power that is much higher when compared to other conventional clinical imaging techniques such as ultrasound, X-ray tomography, and magnetic resonance imaging. Optical microscopy is able to provide resolution in the submicron range, enabling it to resolve fine structures inside tissues. Its application has been extended into neural science which successfully shows sensory information within the rat brain in distributed spatiotemporal patterns of neuronal activity^1^. Although capable of providing high-resolution parameters, its clinical application is still limited by the imaging depth. As light scattering and absorption introduce both light pulse broadening, defocusing, and decrement of intensity, optical image quality and contrast will be compromised with increasing imaging depth. In order to overcome the depth limitation and to enhance the image quality, researchers have attempted to reduce the scattering of tissue turbidity by matching the refractive index using various osmotic agents, a procedure known as optical clearing (OC).

OC was first studied by W Spalteholz in 1914^2^. For the case of skin, Tuchin first studied OC with the tissue immersion technique in 1997^3^. Since then, OC has been frequently studied with various tissues and different optical clearing agents (OCA). One of the most important mechanisms is the refractive index matching, which is to reducing the scattering inside the tissue. However, in different tissues such as sclera^3^, liver^4–6^, muscle^7^, tendon^5,7^, skin^5,8–21^, the OC effect varies. Among these studies, skin optical clearing with glycerol attracts high attention^5,8–15,17,18,20–37^. Glycerol is a low toxic substance and widely used for the food industry, medicine, and cosmetic products. Previous investigations illustrate that after applying glycerol cream or solutions on skin, the skin condition will be improved^38,39^. Glycerol is also one of the most widely used OCAs and has been proved as the most effective among different kinds of alcohols^9,20^. Skin is the outermost organ of humans, which acts as the barrier between internal organs and the environment. Many skin diseases can be histopathologically diagnosed by observing morphological changes in different skin layers. However, the skin is a high scattering tissue that comprises multiple layers with a variation of refractive index. Although OC of human skin have been studied by many different optical imaging techniques such as near-infrared (NIR) spectrophotometer^9–12,23,29,36^, confocal microscopy^13,14^, multi-photon microscopy^8,15,35^, SHG microscopy^8^, and optical coherence tomography (OCT)^5,12,17,18,26,29,30,34,36,37^ in the past few decades, the effects and mechanisms of optical clearing in human skin with glycerol topically applied are not fully understood and are with inconsistent results, especially for the case of glycerol at low concentration. Observation of the OC effect inside the topmost epidermis layer is thus important to understand its mechanisms for topical applications.

In this article, we report our OC study on ex vivo human skin with 50% glycerol application by harmonic generation microscopy (HGM). By adopting a femtosecond excitation light^40^ located at the skin penetration window around 1300 nm^41^, HGM has been confirmed to be a noninvasive histopathology imaging tool for the differential diagnosis of various skin lesions^42–46^, the study of skin aging^10,47,48^, and other clinical tissues^49–52^. For comparison purpose, we applied 100% or 50% glycerol by immersion or topical application on human skin to observe if the OC effect varied. We studied the 100% glycerol with immersion due to its well-understood and promising effects. For the 50% glycerol with topical application, 50% glycerol is safe for in vivo human skin applications and been allowed as human skincare products. The safety assessment of The Cosmetic Ingredient Review Expert Panel (CIR) reported that glycerin can be used at concentrations up to 78.5% in leave-on products^53^. The results of 50% glycerol topical application will help future in vivo human skin trials. Our HGM study indicated the capability of THG microscopy to observe different diffusion effects of OCAs due to its high 3D resolution and high sensitivity to the local change in optical homogeneity in epidermis. SHG was, on the other hand, a beacon to summarize the accumulated reduction of the optical scattering in the epidermis. Our study revealed that both refractive index matching and tissue shrinkage affect the optical clearing. Besides, we found the stratum corneum (SC) playing an important role for topical application of OCA. Among all the studied 9 cases, six cases found decrement of the SC thickness, accompanying significant optical clearing effects. The HGM intensity, either THG or SHG, at the epidermis or dermis would be enhanced due to the reduction of the light scattering in SC. Our study also attributes different optical clearing effects of 100% and 50% glycerol to different resulting homogeneities as well as dissimilar diffusion depths.

## Results

### Optical clearing effect of 100% glycerol immersion

**Figure 1:**
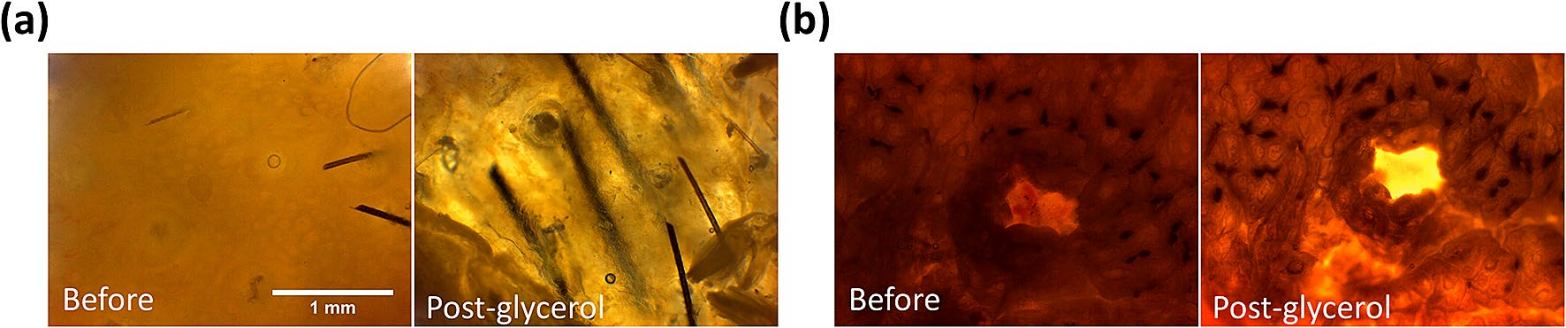
Bright field images before and after the glycerol application of the (**a**) Case E100-I90-1 (**b**) Case E100-I90-2. Scale bar = 1 mm. Before: before treatment; Post-glycerol: after treatment.

First, we discuss the results of Case E100-I90, which was under the most frequently studied condition by using 100% glycerol immersion of 90 min. Two skin tissues were studied, named Case E100-I90-1 (skin of scalp) and Case E100-I90-2 (skin of armpit). After the glycerol application, the transparency of the skin was highly enhanced, and the hair inside the skin became visible (Fig. 1).

**Figure 2:**
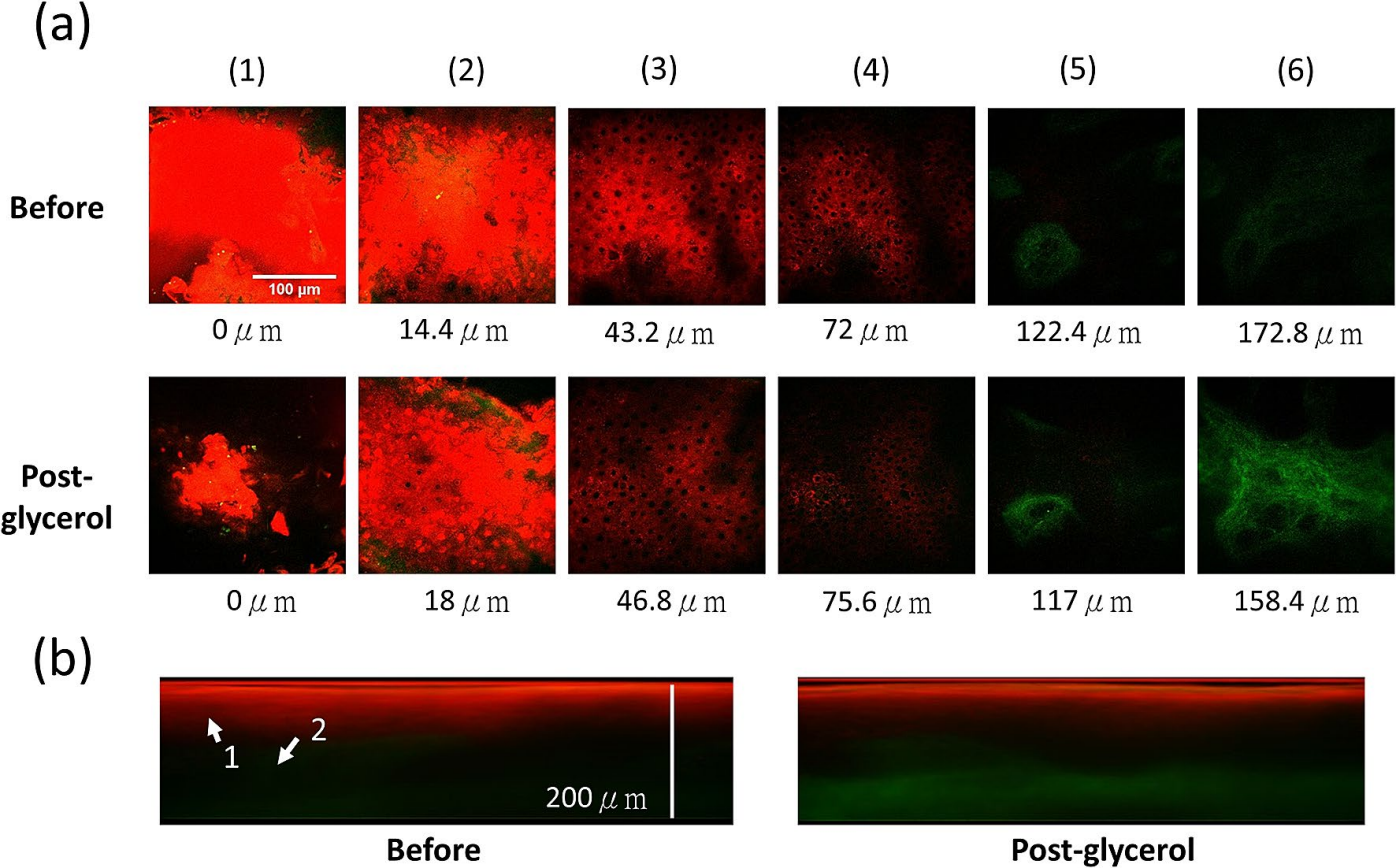
Ex vivo HGM images of Case E100-I90-1, with 100% glycerol immersion. (**a**) *En face* HGM images of (1) Top layer of the SC. (2) Bottom layer of the SC. (3) Middle layer of the viable epidermis. (4) Basal layer. (5) Middle layer of the papillary dermis. (6) Top layer of the reticular dermis. Scale bar = 100 μm. (**b**) Transverse HGM images. The significant depth difference of the reticular dermis layer is attributed to the sample shrinkage post glycerol immersion. Arrow 1: Epidermis; Arrow 2: Dermis.

**Figure 3:**
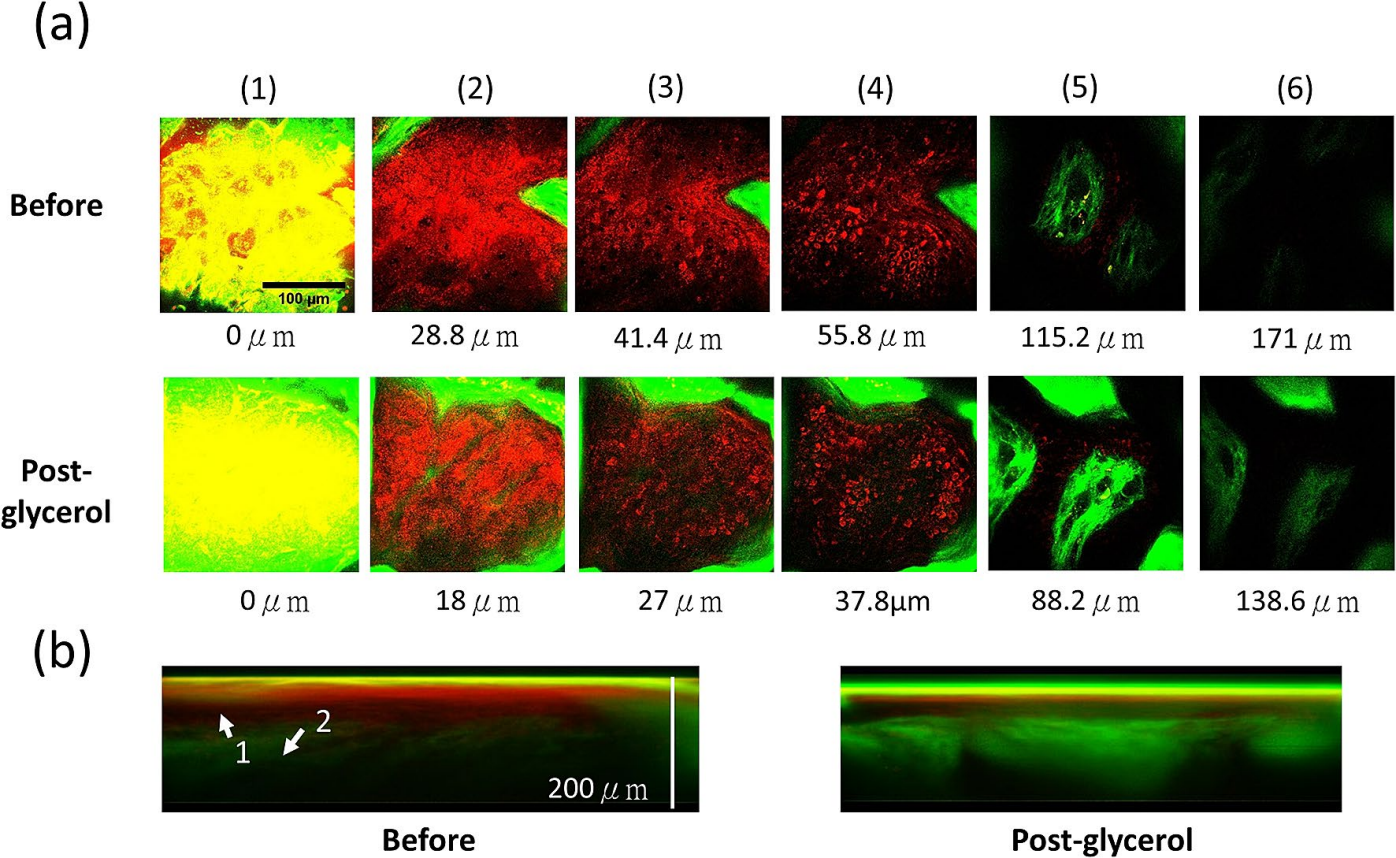
Ex vivo HGM images of Case E100-I90-2, with 100% glycerol immersion. (**a**) *En face* HGM images of (1) Top layer of the SC, (2) Bottom layer of the SC, (3) Middle layer of the viable epidermis, (4) Basal layer, (5) Middle layer of the papillary dermis, and (6) Top layer of the reticular dermis. Scale bar = 100 μm. (**b**) Transverse HGM images. In the layer of SC, the strong two-photon fluorescence leakage of the residue marking dye into the SHG channel is observable and is not included in our SHG analysis. The significant depth difference of the corresponding layer is attributed to the sample shrinkage post glycerol immersion. Arrow 1: Epidermis; Arrow 2: Dermis.

Figures 2 and 3 showed representative HGM stack images at different skin layers before (Before) and after (Post-glycerol) the glycerol application. In both cases, the THG (red) intensity and contrast in the epidermis decreased. While in the dermis, the SHG (green) intensity of the collagen strongly increased^54^. Similar to the Case E100-I90-1, the THG provided cell structure at the viable epidermis of the Case E100-I90-2 became less visible after the glycerol application, in contrast to the much-enhanced SHG intensity in the dermis^54^. The green signal at the sample surface was attributed to autofluorescence leakage due to residual dye from a marker pen.

**Figure 4:**
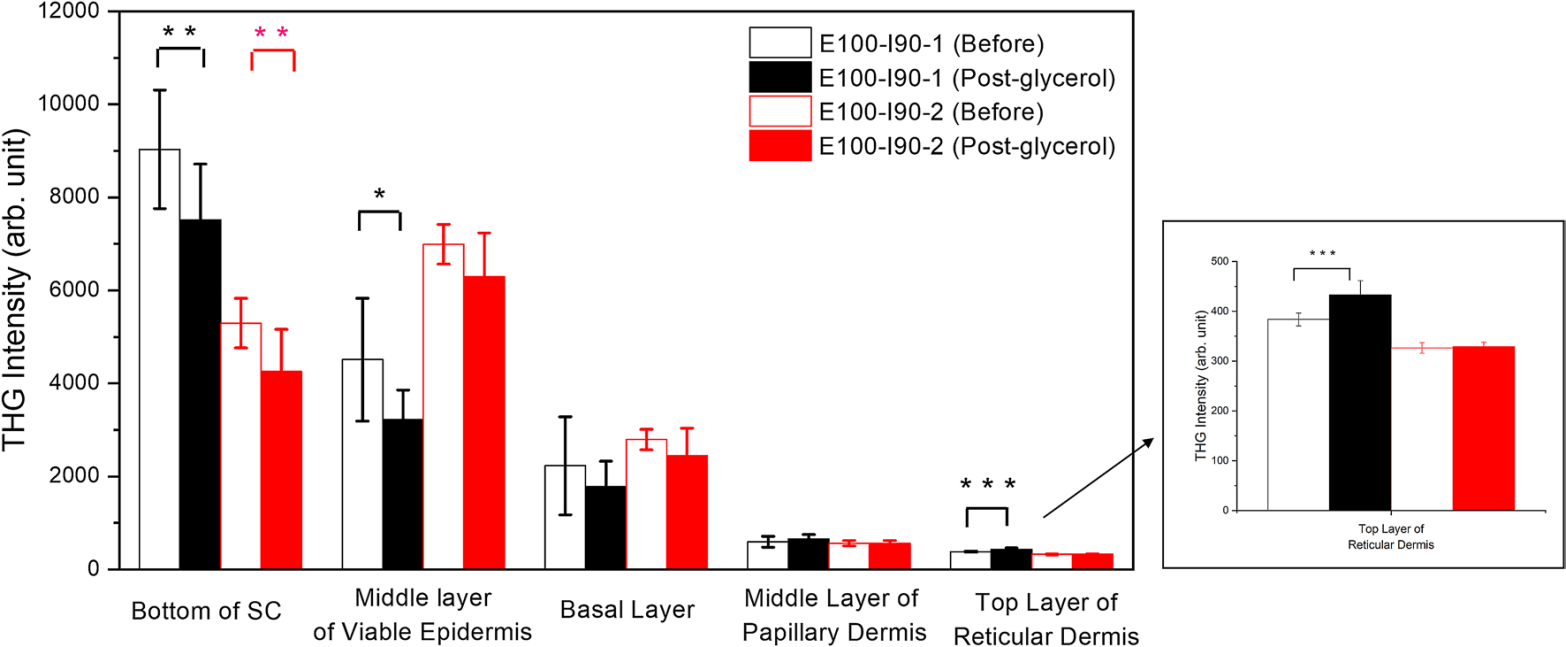
Quantitative analysis and comparison of skin THG intensities at five different depths. Black: Case E100-I90-1 (Before: N = 8, Post-glycerol: N = 8); Red: Case E100-I90-2 (Before: N = 9, Post-glycerol: N = 9). Inset shows an enlarged view of the statistical result. *P < 0.05; **P < 0.01; *** *P* < 0.001. Before: before treatment; Post-glycerol: after treatment.

**Figure 5:**
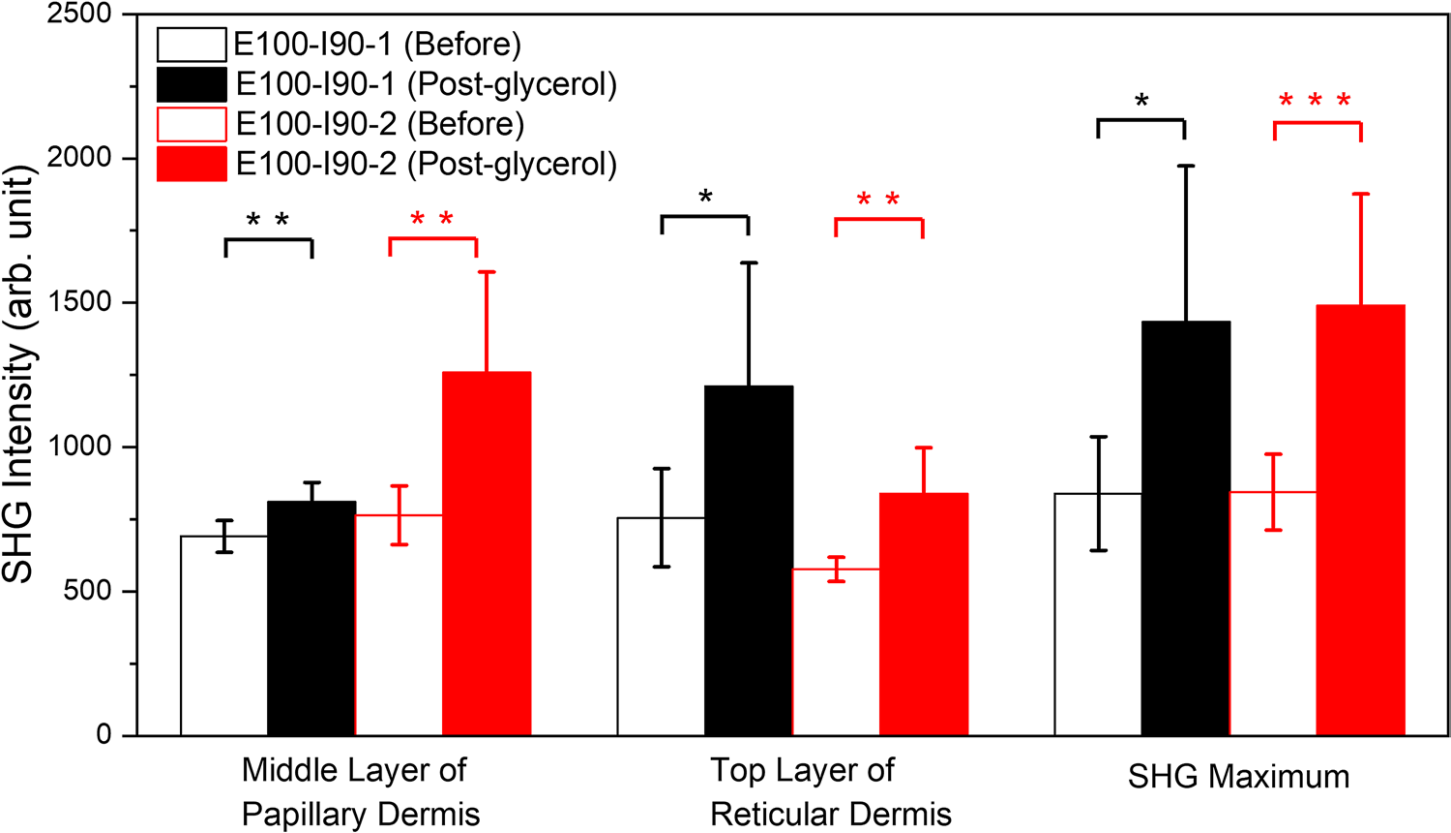
Quantitative analysis and comparison of skin SHG intensities at three different depths. Black: Case E100-I90-1 (Before: N = 8, Post-glycerol: N = 8); Red: Case E100-I90-2 (Before: N = 9, Post-glycerol: N = 9). *P < 0.05; ** *P* < 0.01; *** *P* < 0.001. Before: before treatment; Post-glycerol: after treatment.

Figures 4 and 5 show the statistical analysis of the THG and SHG intensities in Case E100-I90. For THG, the average intensities reduced significantly at superficial levels (SC, and the viable epidermis) but increased significantly at deep levels (the reticular dermis) in Case E100-I90-1. While in Case E100-I90-2, the average THG intensities reduced significantly only at SC. For SHG, the average SHG intensities increased at all three positions in both Case E100-I90-1 and Case E100-I90-2.

Table 1 recorded the structure analysis results of all studied cases. For Case E100-I90-1, after 100% glycerol immersion, only the depth of the top reticular dermis layer was found with a significant difference, which increased by only 7%. In contrast, in the Case E100-I90-2, the skin shrank significantly at all layers. In previous studies, the anhydrous glycerol was proved to make the skin shrink^9,10,12^. However, we only found significant shrinkage of the skin in Case E100-I90-2. The skin of the armpit was softer than the skin of the scalp to make it shrink more severely.

**Table 1:** Skin structure analysis results of all studied cases. (*P < 0.05, ** *P* < 0.01, *** *P* < 0.001).

	Depth or thickness change (μm)	Thickness of SC	Thickness of viable epidermis	Depth of basal layer	Depth of middle papillary dermis layer	Depth of top reticular dermis layer
**Case E100-I90 (immersion in 100% glycerol for 90 min)**						
1	Before (N = 8)	13.7 $\pm$ 4.6	54.9 $\pm$ 13.4	68.6 $\pm$ 13.0	113.0 $\pm$ 9.1	156.6 $\pm$ 8.8
	Post-glycerol (N = 8)	12.6 $\pm$ 4.6	50.6 $\pm$ 7.5	63.2 $\pm$ 11.6	116.1 $\pm$ 7.8	168.5 $\pm$ 13.4*
2	Before (N = 9)	30.0 $\pm$ 2.4	29.2 $\pm$ 3.5	59.2 $\pm$ 3.9	114.2 $\pm$ 6.7	167.8 $\pm$ 13.3
	Post-glycerol (N = 9)	21.6 $\pm$ 4.5***	20.4 $\pm$ 5.3***	42.0 $\pm$ 4.2***	87.0 $\pm$ 5.2***	131.0 $\pm$ 10.5***
**Case E50-I90 (immersion in 50% glycerol for 90 min)**						
1	Before (N = 6)	12.0 $\pm$ 3.4	15.3 $\pm$ 3.7	27.3 $\pm$ 4.8	66.6 $\pm$ 10.4	108.0 $\pm$ 30.9
	Post-glycerol (N = 6)	7.5 $\pm$ 1.4*	15.0 $\pm$ 5.7	22.5 $\pm$ 5.4	66.0 $\pm$ 16.9	105.0 $\pm$ 16.1
2	Before (N = 12)	30.6 $\pm$ 3.0	27.8 $\pm$ 5.8	58.4 $\pm$ 4.5	109.1 $\pm$ 5.1	158.7 $\pm$ 10.1
	Post-glycerol (N = 12)	18.0 $\pm$ 3.5***	35.1 $\pm$ 6.8**	53.1 $\pm$ 8.3	120.8 $\pm$ 4.4***	187.7 $\pm$ 6.0***
**Case E50-T90 (topical applied 50% glycerol for 90 min)**						
1	Before (N = 8)	22.1 $\pm$ 4.8	33.8 $\pm$ 6.1	55.8 $\pm$ 6.3	95.4 $\pm$ 5.4	134.1 $\pm$ 12.7
	Post-glycerol (N = 8)	13.3 $\pm$ 1.6***	42.3 $\pm$ 6.5*	55.6 $\pm$ 7.0	94.1 $\pm$ 9.0	132.3 $\pm$ 12.6
2	Before (N = 10)	33.3 $\pm$ 4.6	34.9 $\pm$ 6.2	68.2 $\pm$ 4.8	122.9 $\pm$ 9.2	176.4 $\pm$ 17.3
	Post-glycerol (N = 10)	18.0 $\pm$ 3.1***	40.1 $\pm$ 6.0	58.1 $\pm$ 5.8***	120.8 $\pm$ 5.7	182.5 $\pm$ 9.7
3	Before (N = 9)	12.2 $\pm$ 2.0	53.8 $\pm$ 12.4	66.0 $\pm$ 10.8	93.4 $\pm$ 15.1	120.4 $\pm 13.7$
	Post-glycerol (N = 9)	13.0 $\pm$ 1.5	57.2 $\pm$ 7.8	70.2 $\pm$ 8.3	92.2 $\pm$ 12.2	113.4 $\pm$ 17.6
4	Before (N = 7)	27.3 $\pm$ 9.0	63.2 $\pm$ 8.1	90.5 $\pm$ 6.8	116.8 $\pm$ 8.6	145.0 $\pm$ 15.9
	Post-glycerol (N = 7)	15.1 $\pm$ 2.8***	55.5 $\pm$ 7.0*	70.5 $\pm$ 6.8***	102.6 $\pm$ 7.8***	133.5 $\pm$ 15.1

Besides Table 1, we summarize all the statistical analysis results on the THG and SHG image intensities in Tables 2 and 3, separately.

**Table 2:** Statistical analysis result on the THG intensity variation after the glycerol application.

THG intensity	Bottom of SC	Middle viable epidermis layer	Basal layer	Middle papillary dermis layer	Top reticular dermis layer
**Case**	**E100-I90 (100% Immersion)**				
1	▽ (**)	▽ (*)	–	–	△ (***)
2	▽ (**)	–	–	–	–
**Case**	**E50-I90 (50% Immersion)**				
1	–	–	–	△ (**)	△ (*)
2	△ (***)	△ (***)	△ (**)	△ (**)	–
**Case**	**E50-T90 (50% Topical Application)**				
1	–	–	–	–	–
2	△ (***)	△ (*)	△ (*)	△ (*)	–
3	–	–	–	△ (*)	–
4	△ (***)	△ (***)	△ (***)	–	–

−: no significant difference, △: Increased significantly, ▽: Decreased significantly, *P < 0.05, ** *P* < 0.01, *** *P* < 0.001.

**Table 3:** Statistical analysis result on the SHG intensity variation after the glycerol application.

SHG intensity	Middle papillary dermis layer	Top reticular dermis layer	SHG Maximum
**Case**	**E100-I90 (100% Immersion)**		
1	△ (**)	△ (*)	△ (*)
2	△ (**)	△ (**)	△ (***)
**Case**	**E50-I90 (50% Immersion)**		
1	△ (*)	△ (*)	△ (*)
2	△ (*)	△ (***)	△ (***)
**Case**	**E50-T90 (50% Topical Application)**		
1	–	△ (*)	△ (*)
2	–	△ (*)	△ (*)
3	–	–	–
4	–	–	–

−: no significant difference, △: Increased significantly, ▽: Decreased significantly, *P < 0.05, ** *P* < 0.01, *** *P* < 0.001.

From the bright-field images, we observed that after glycerol application the skin became transparent and the hair inside the deep area was more visible in both skin tissues (Fig. 1). Glycerol is with a higher refractive index (1.47) than extracellular and intracellular fluids (1.34–1.36). After immersion the skin tissues into anhydrous glycerol, glycerol diffused inside and filled all of the skin tissues to create a higher refractive index matching environment for the scattering particles (such as organelles, protein fibrils, membranes, protein globules, with refractive index 1.39–1.47^55,56^). Therefore, the light scattering was reduced and the image depth increased. This result is similar to and supported by the previous research of G. Vargas et al. in 1999, by observing the rat skin after the glycerol application^12^. By using spectrophotometer and OCT they proved that glycerol will diffuse into the skin to decrease the refractive index mismatch. This result also correlates with many previous studies^8,18,57^.

With much-reduced scattering and higher transparency in the studied samples, one expects an obvious increase of the SHG intensity at deeper dermis layers. Scattering in skin tissues will not only results in a decrease of excitation intensity, but also defocus the point spread function. The combined effect will not only cause SHG and THG signals to decrease, but will also degrade the system resolution and contrast with increased imaging depth^58^. As a result, in both samples of 100% glycerol immersion, we witnessed that the SHG image intensity in the dermis was greatly enhanced after the optical clearing. Combining with the observed higher sample transparency, we attributed the SHG enhancement to the effect that the excitation light was able to travel down to deep regions of the skin with less energy decrement and less wavefront distortion. Moreover, the reduction of scattering also makes it easier for SHG signals to travel back to the detector. Our SHG results correlate well with previous OC studies.

Besides excitation intensity and point spread function^58^, THG intensities also strongly depend on the refractive index mismatch inside the excitation focus^59,60^. After the glycerol application, the OC effect will not only restore the excitation light and allow easier collection of the generated nonlinear signals, as been indicated by the SHG intensity recovery, but will also decrease the refractive index mismatch. These two different mechanisms will compete for the THG intensity. For 100% glycerol immersion cases, THG intensity decreased in epidermis while increased or remain the same in dermis, indicating that refractive index matching is the dominant effect in epidermis to determine the THG intensity due to the shallow penetration depth. Although glycerol will greatly enhance the homogeneity of the tissue and reduce the THG radiation, in the deeper layer like dermis, a strong reduction of scattering of the whole epidermis will result in a strong increment of excitation intensity and thus eventually the detected THG photons. As a result, the THG image intensity increased or remained the same in dermis after optical clearing.

Dehydration and tissue shrinkage were frequently observed during optical clearing in previous studies^55,61,62^. In Case E100-I90-2, tissue shrinkage was observed. It is known that the strong affinity of glycerol would make the water flux out tissues and lead to dehydration, with different permeability coefficients of water and glycerol, which are on the order of ${10}^{-2}$ cm/min and ${10}^{-5}$ cm/min, respectively. This difference made more water flux out than glycerol enter the skin, causing the tissue volume decrease^32^. By moving the skin structure toward the surface due to shrinkage, one could also expect to see improved image intensities.

### Optical clearing effect of 50% glycerol immersion

**Figure 6:**
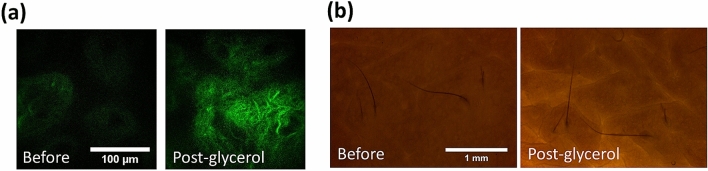
Optical clearing with 50% glycerol immersion. (**a**) SHG images of dermis (scale bar = 100 μm), and (**b**) bright field images. (scale bar = 1 mm). This example belongs to Case E50-I90-2. Before: before immersion; Post-glycerol: after immersion.

In the case of 50% glycerol application with immersion of 90 min, one might expect a lower OC effect due to the lower glycerol concentration. Under the bright light microscopy (Fig. 6), the sample transparency after 50% glycerol immersion was less than that after 100% glycerol immersion. On the other hand, although with its lower refractive index (1.398)^63^ and lower dehydration power compared to 100% glycerol, we found that the 50% glycerol was also able to decrease the scattering in the tissue, evidenced not only by the improved transparency after immersion, but also by the significantly increased SHG intensity in dermis (Fig. 6). Our studies of two samples indicated greatly enhanced SHG intensities in dermis after the optical clearing (Table 3).

**Figure 7:**
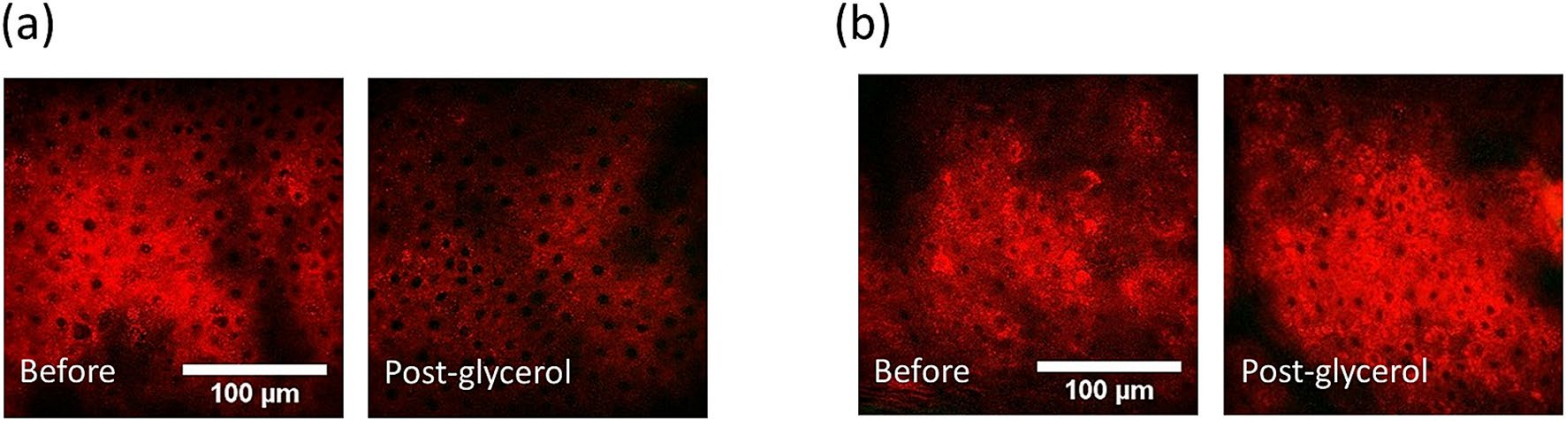
THG intensity variation at the middle epidermis layer of (**a**) Case E100-I90-1 with 100% glycerol and (**b**) Case E50-I90-2 with 50% glycerol. Before: before immersion; Post-glycerol: after immersion. Scale bar = 100 μm.

The most significant difference between these two different concentrations is the variation of the THG intensity, as shown in Fig. 7. In the middle epidermis layer, for a case of 100% glycerol immersion (Case E100-I90-1), the average THG intensity reduced 13%. However, for a specific case of 50% glycerol immersion (Case E50-I90-2), the average THG intensity increased 30%. Different from all cases of 100% glycerol immersion, of which the THG intensity greatly decreased in the epidermis after OC, the THG intensity of all 50% immersion cases either increased or remained the same (Table 2). As discussed above, the THG intensity is affected by the refractive index mismatch inside the excitation focus^59,60^. The 50% glycerol has a lower osmolarity than 100% glycerol^8,63^ and cannot introduce such a high homogeneous environment as the 100% glycerol. The optical clearing power of 50% glycerol is also known to be less than the 100% glycerol. As a result, for 50% glycerol immersion, there was a compromise for THG intensity between the decrement of the light scattering and the decrement of the refractive index mismatch. The less decrement of refractive index mismatch and the recovery of the light intensity and quality made the THG intensity enhanced or remain the same after the OC. Besides, in both two tissues of the 50% immersion cases, the thickness of the SC all greatly decreased by at least 37.5%, which would also make the detection of THG signal easier. In the skin tissue with thicker SC, Case E50-I90-2, the THG intensity even increased in dermis. To sum up, by immersed the skin tissue inside the 50% glycerol, we could not only enhance the SHG intensity but also the THG intensity and the THG image in epidermis can be better resolved.

### Optical clearing effect of 50% glycerol topical application

**Figure 8:**
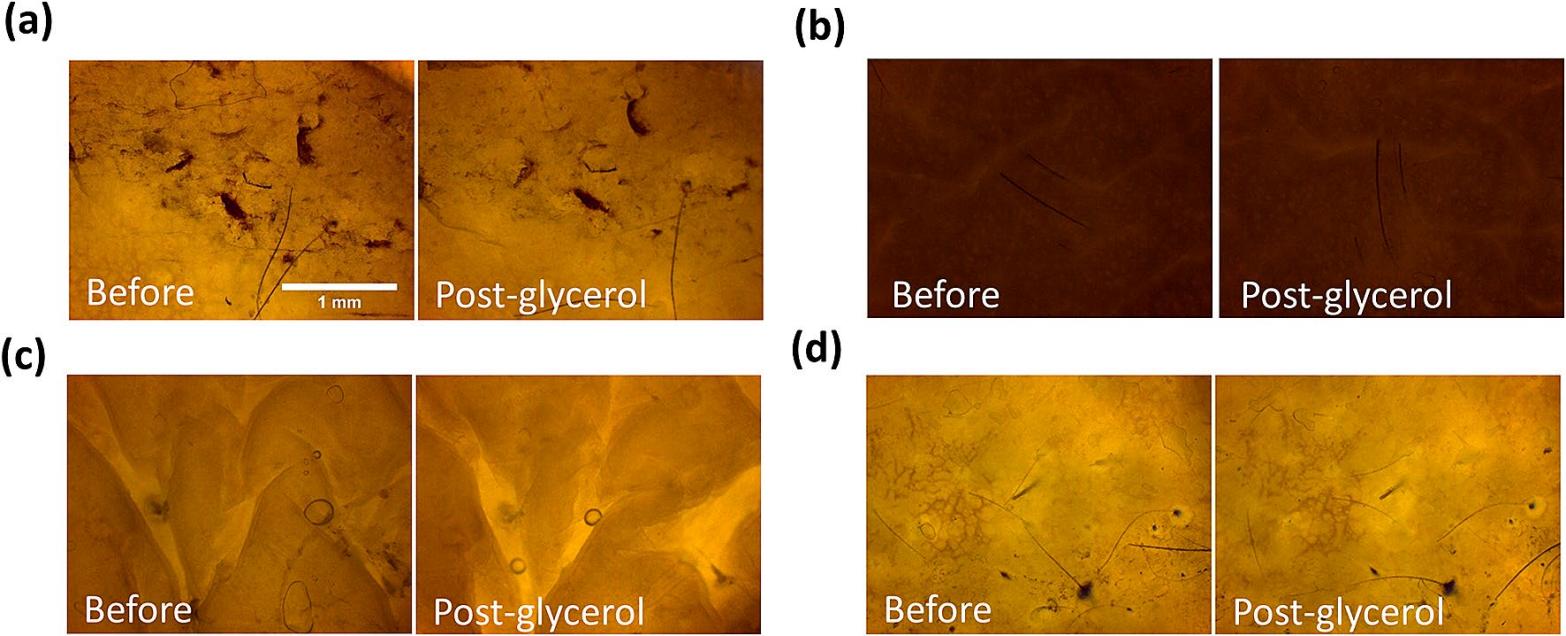
Bright field images of human skin before (Before) and after (Post-glycerol) 50% glycerol topical application. (**a**) Case E50-T90-1 (**b**) Case E50-T90-2 (**c**) Case E50-T90-3 (**d**) Case E50-T90-4. Scale bar = 1 mm.

To study the OC potential for in vivo clinical applications, the final aim of this work is to investigate the OC effect of ex vivo human skin by using 50% glycerol with noninvasive topical application, while immersion studies above served as calibration with previous reports. Four skin tissues from different volunteers were included, which were the skin of forehead, buttock, chest, and temporal. After the 50% glycerol topical application, only two tissues were found with the statistically-significant SHG enhancement in dermis (forehead, buttock; Table 3). On the other hand, the average SHG in all tissues were found to be higher after the topical applications, even without statistical significance. As a result, the signal recovery effect of 50% glycerol topical application is less than the 50% and 100% glycerol immersion. In the bright light image, the transparency of all four skin tissues was slightly improved, showing less decrement of the scattering when compared to the immersion case with 50% glycerol (Fig. 8) and confirming our SHG finding.

Our study supports the OC effect through 50% glycerol topical application. As in comparison with the immersion case, which is impossible for in vivo clinical applications, the clinical applicable topical application is with a less effective OC effect. In the case of topical application, the glycerol solution can diffuse into the epidermis only through the barrier function of SC, which greatly reduces the penetration of the glycerol solution. As we did not observe any SC-thickness (before application) dependence (Table 1) on the SHG enhancement, the sample-dependent OC results might come from: (1) Variation of the SC permeability at different parts of the skin^64–66^; (2) Different skin conditions of different volunteers, therefore causing different skin barrier functions. On the other hand, we did observe one interesting phenomenon related to SC, that is, among all four tissue samples, three were found to be with a much decreased (at least 40%) SC thickness, including cases 1, 2, and 4. The only exception sample was with an extremely thin SC layer (12 μm-thick), which left no room for further shrinkage after OCA application. For these three SC-shrinking samples, we either observed recovered SHG signals in dermis, or recovered THG signals in epidermis, indicating the critical role of SC during optical clearing after topical application. We thus propose that if one can reduce the thickness of SC through OC, the SHG or THG intensity underneath the SC layer will then have a potential to be rescued. Our study further supports that with 50% glycerol topical application of 90 min, one is capable to decrease the thickness of SC, if the thickness of SC is greater than 22 μm.

**Figure 9:**
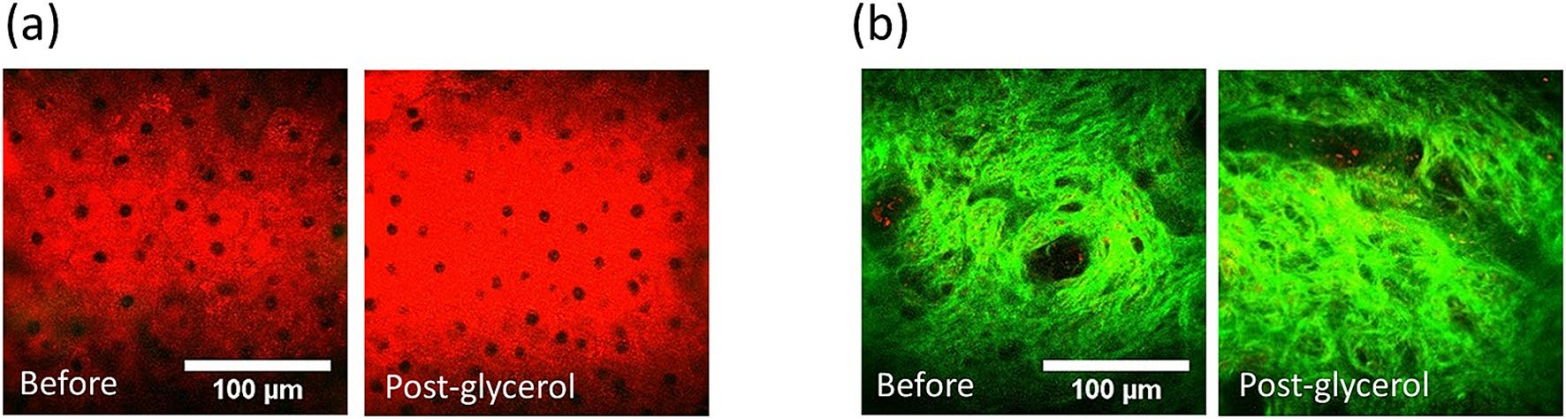
(**a**) The variation of the THG image intensity at middle epidermis layer after 100% glycerol topical application for 30 min. (**b**) The variation of the SHG image intensity at top reticular dermis layer after 100% glycerol topical application for 30 min. Before: before treatment; Post-glycerol: after treatment. Scale bar = 100 μm.

In contrast to the fact that there is no SC-thickness dependence (Table 1) on the SHG enhancement, in our studied cases by using 50% glycerol with either immersion or topical application, we found SC-thickness dependence (Table 1) on the THG enhancement in epidermis. The epidermal THG signal was increased after 50% glycerol application for all samples with a SC layer thicker than 27 μm, including cases E50-I90-2, E50-T90-2, and E50-T90-4. This might be due to the fact that the skin tissue with a thick SC layer suffers stronger light scattering so that the OC effect is more pronounced. Our observation also suggests the important role of SC to affect the epidermal THG signal after OC. To further investigate the role of SC in OC effects, we additionally performed an OC experiment by using 100% glycerol with topical application. In previous studies, high concentration glycerol was proved to be with a slower permeation rate than lower concentration glycerol^8^. It can be described by the Fick’s law, in which the skin permeability is inversely proportional to the glycerol concentration^17^. Based on the lower penetration rate with its higher viscosity and the stronger OC effect as compared to the 50% glycerol, the 100% glycerol was applied on the skin surface for 30 min, in contrast to the 90 min, to provide the OC effect in SC only (named: Case E100-T30). The results of the Case E100-T30 were presented in Table 4. Representative HGM skin images were presented in Fig. 9.

**Table 4:** Variation of skin structure, THG intensity, and SHG intensity after 100% glycerol topical application for 30 min.

Case E100-T30					
Depth or thickness (μm)	Thickness of SC	Thickness of viable epidermis	Depth of basal layer	Depth of middle papillary dermis layer	Depth of Top reticular dermis layer
Before (N = 10)	13.5 $\pm$ 0.9	17.3 $\pm$ 4.3	30.8 $\pm$ 4.7	60.1 $\pm$ 6.0	88.7 $\pm$ 13.2
Post-glycerol (N = 10)	13.5 $\pm$ 2.6	21.6 $\pm$ 2.4*	35.1 $\pm$ 3.3*	67.0 $\pm$ 8.0*	98.1 $\pm$ 16.9

THG intensity	Bottom of SC	Middle viable epidermis layer	Basal layer	Middle papillary dermis layer	Top reticular dermis layer
After the applying glycerol	△ (***)	△ (***)	△ (***)	△ (***)	–

SHG intensity	Middle papillary dermis layer	Top reticular dermis layer	SHG maximum		
After the applying glycerol	△ (*)	–	–		

−: no significant difference, △: Increased significantly, ▽: Decreased significantly, *P < 0.05, ** *P* < 0.01, *** *P* < 0.001.

Our result supports the OC effect through 100% glycerol topical application. The average THG intensities greatly increased at all skin layers except the top reticular dermis layer. For SHG, the average SHG intensity of the papillary dermis also showed significant increment (Table 4). Even though with the method of the topical application, 100% glycerol solution cannot diffuse as far as the immersion case and the optical clearing is less effective, 100% glycerol seems to provide a better SC effect of thin SC layer, in comparison with 50% glycerol, thus increasing the laser excitation power in layers beneath as well as the back-reflection signals. The fact that glycerol did not reach the viable epidermis can be evidenced by the enhanced THG intensities through the viable epidermis.

## Discussions

The aim of the current paper is to study the optical clearing effect of 50% glycerol through topical application due to the fact that 50% glycerol, rather than 100% glycerol, is safe for in vivo human skin applications and been allowed as human skincare products. During our study, we did not remove the stratum corneum layer. As a result, we found the critical role of stratum corneum shrinkage for the recovery of the SHG and THG images in human skin. Optical clearing of human skin has been widely studied in previous papers. Some of the papers focus on comparing different OCAs, the others investigate the mechanisms behind the OC. Most of the previous studies normalize the image intensity to determine the penetration depth^17,34^ and compares the average intensity before and after OCA application^8,30^, few of them adopted the statistical analysis like in this study. However, the conclusion might be different under different analysis procedures. For example, in this study, it is noted that the mean SHG intensities all increase after OC at the middle papillary dermis layer, top reticular dermis layer, and SHG maximum layer in all studied 9 cases, even though some of increment might not be statistically significant. The mean SHG intensity of all studied cases is shown in Table 5.

**Table 5:** Mean SHG intensities (arbitrary unit) of all studied cases at the middle papillary dermis layer, top reticular dermis layer, and SHG maximum layer. (*P < 0.05, ** P < 0.01, *** P < 0.001).

Case number	Group	Middle papillary dermis layer	Top reticular dermis layer	SHG Maximum
**Case E100-I90**				
1	Before (N = 8)	691	756	839
	Post-glycerol (N = 8)	810**	1210*	1434*
2	Before (N = 9)	764	577	844
	Post-glycerol (N = 9)	1258**	839**	1490***
**Case E50-I90**				
1	Before (N = 6)	1156	957	1246
	Post-glycerol (N = 6)	2552*	3918*	4093*
2	Before (N = 12)	768	930	1002
	Post-glycerol (N = 12)	887*	1386***	1444***
**Case E50-T90**				
1	Before (N = 8)	966	1522	1702
	Post-glycerol (N = 8)	1092	2346*	2594*
2	Before (N = 10)	711	795	841
	Post-glycerol (N = 10)	755	1070	1141
3	Before (N = 9)	1411	1547*	1701*
	Post-glycerol (N = 9)	1458	1819	1909
4	Before (N = 7)	949	1377	1419
	Post-glycerol (N = 7)	1079	1493	1547
**Case E100-T30**				
1	Before (N = 10)	2366	3692	3789
	Post-glycerol (N = 10)	3138*	4050	4362

Although OC have been investigated by many previous studies, the effects and the mechanisms of 50% glycerol after topical application in human skin are not well understood. In this paper, we first combined the SHG and THG microscopy images to study the OC effect of skin. The ultrahigh spatial resolution of HGM provided more details regarding the skin OC effect.

In our studied case of 100% glycerol immersion, the glycerol diffused into the skin and made it more homogeneous to reduce the light scattering. The THG intensity depends strongly on refractive index mismatch and will vanish in homogeneous media. The 100% glycerol greatly enhanced the tissue homogeneity and the observed THG intensities were therefore decreased. With a better epidermal tissue homogeneity and reduced scattering reduction, we also observed enhanced SHG intensities in the underlying dermis. We attributed the enhanced SHG in dermis to the OC effect in epidermis. It is noted that in a previous paper, decreased SHG intensity was reported after OC^67^. Different from our experimental condition, Yeh et al. studied the rodent skin by removing the epidermis and then applying glycerol. They concluded that the reversible dissociative effect by glycerol on collagen may be one of the mechanisms of OC. We regard that the main mechanism in our case is different from Yeh since we preserve the complete epidermis. Furthermore, we did not find the dissociation of collagen in our HGM observation. We also studied the cases of the 50% glycerol by immersion or topical application since it is a safe concentration to apply on the human skin. In both methods, we observed the enhancement of the SHG intensities, rather than decrement, but the OC is less effective by topical application.

Three OC mechanisms were mentioned in previous studies: the refractive index matching^8,18,57^, the dehydration^5,10,11,55^, and the shrinkage of the tissue^11,32^. In our research, we found that the refractive index matching and the tissue shrinkage are the main mechanisms affecting the optical clearing efficacy. Besides, the stratum corneum plays an important role to achieve the optical clearing. For THG signal enhancement in the epidermis, SC acts the most important role. After the topical applications with the 50% glycerol, we observed stronger OC effect in thicker SC skin tissues, accompanying significant SC shrinkage. In conclusion, we achieve the optical clearing of human skin by using the 50% glycerol with the topical application. The results of this study suggest the potential of 50% glycerol for future clinical OC topical applications, especially for skin with a thick SC layer.

## Methods

### Sample preparation

All skin samples were surgical samples from the National Taiwan University Hospital (NTUH). The patients included were aged 20 to 85 years, had Fitzpatrick skin type III or IV. After removing the lesion part of the excised skin, we choose the non-lesional human skin samples of which the remaining surface areas were larger than 0.5*0.5 cm^2^ for experiments. The skin samples were not limited to any specific area of the human body to maximize the study cases. The skin sample were taken from scalp (case E100-I90-1, E50-I90-1), axilla (case E100-I90-2), buttock (case E50-I90-2, E50-T90-2), forehead (case E50-T90-1), chest (case E50-T90-3), and left temporal area (case E50-T90-4). The study protocol and safety risks associated were reviewed and approved by the research ethics committee at NTUH (No. 201804100DINA) and Taiwan Food and Drug Administration (TFDA). All research was performed in accordance with relevant guidelines and regulations. All volunteers participating in the optical clearing experiment provided informed consent.

Fresh samples from the surgery room were received within one hour after the excision and we removed the fat tissues under the dermis. We stored samples in saline and kept it at 4 °C to prevent dehydration as well as to remove blood from the skin surface. Before imaging, we soaked the skin sample in fresh saline and then left it under the room temperature for at least 30 min to standardized the initial conditions of all samples in order to get reproducible results. To study the optical clearing of the fresh skin tissue, we completed all experiments within 24 h after the skin was excised from the patient.

### Optical clearing agents

We chose glycerol as the OCA in our experiments, because it is one of the most popular OCAs in previous studies^11,27,30,32,33,38^. Glycerol was purchased from Sigma-Aldrich (Product Number: G2289) which meets USP testing specifications. For 100% glycerol experiments, we used pure glycerol. For 50% (V/V) glycerol solutions, we mixed pure glycerol with deionized water of the same volume.

### Experimental apparatus

For bright-field imaging, we used LEICA ICC50 HD (bright-field trans-illumination microscopy) to document the visual changes on the skin tissue before and after the optical clearing. The eyepiece for CCD was set on 10×/20 combining with the 4×/0.10 objective lens to provide 40 times magnified skin images. Variation of the sample transmission can be observed by bright-field images.

**Figure 10:**
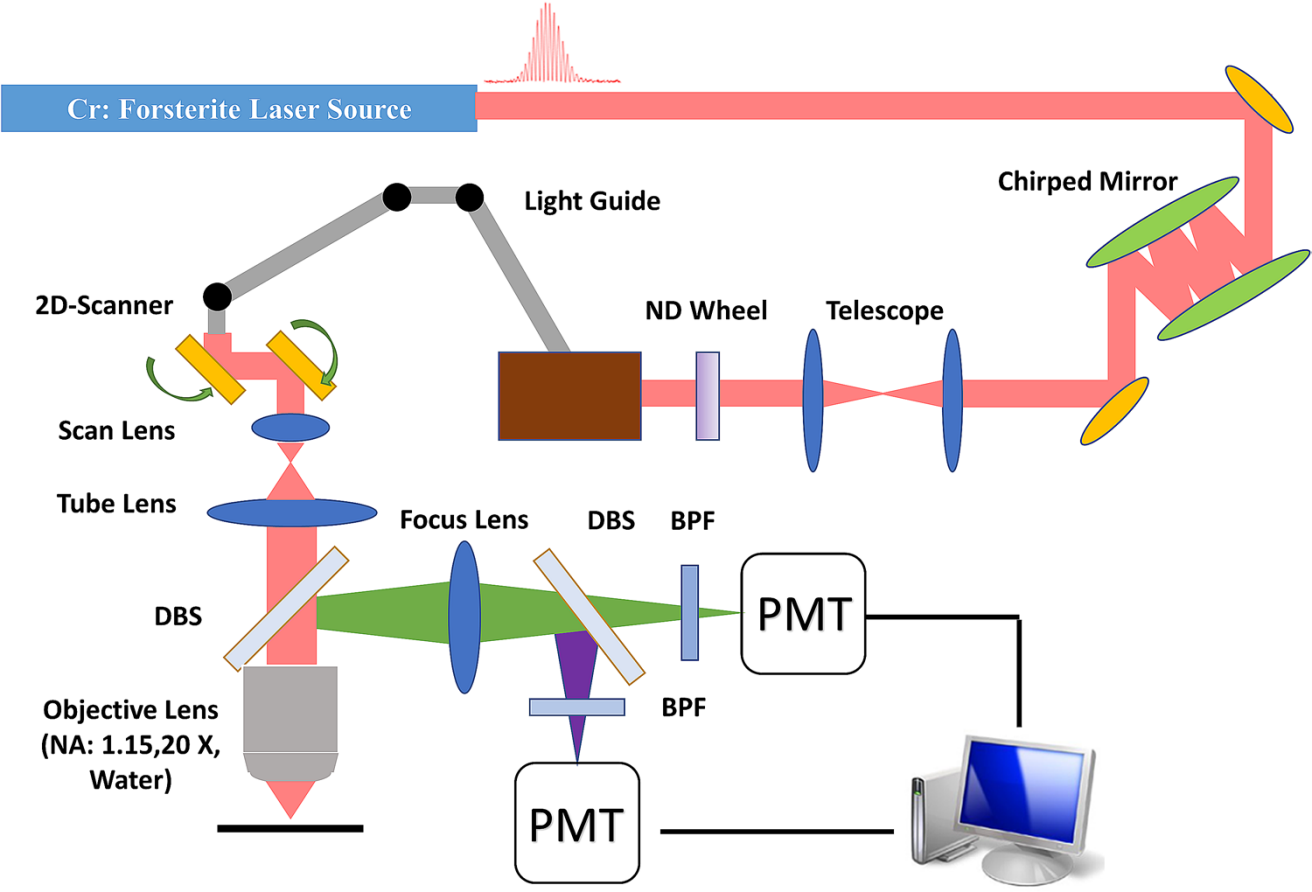
The schematic diagram showing the harmonic generation microscope system. *DBS* Dichroic beam splitter, *BPF* Band-pass filter, *PMT* Photomultiplier tube, *ND* neutral density.

For HGM imaging of human skin, the system setup is shown in Fig. 10. A homemade femtosecond Cr:forsterite laser was used as the light source to provide 1262 nm at the central wavelength^68^. The ultrashort pulse duration (38 fs) of this laser makes it ideal to generate efficient harmonic signals at the focal point. Double chirped mirrors^69^ were utilized to compensate for the dispersion of the optical system for the shortest pulse width in human skin. A telescope (New port, KPX193 AR18) was used to collimate the laser light to maintain the beam size after a long traveling path. The excitation power of the laser can be adjusted and maintained via a ND wheel (New port, 50FS02DV.2). The light guide, which is critical for the human clinical study, was able to move the HGM microscope conveniently to image different areas of the human skin. The 2D scanner was a combination of a resonant 8 kHz mirror and a galvanometer mirror to generate 2D scans (Thorlabs Laser Scanning Essentials Kit). The scan lens and tube lens not only increased the beam size to make it fulfill the back aperture of the objective lens but also formed a 4f system to make tilted scanning light go back to the back aperture of the objective lens. The laser light was focused in the human skin by an objective lens (Olympus, UApoN340, NA = 1.15). The SHG (630 nm) and THG (410 nm) signal would be generated at the focal point and the signal will be collected by the same objective lens. The first dichroic beam splitter (DBS) (Thorlabs/ DMLP900L) was used to reflect the harmonic signals collected by the objective lens and the second DBS (Thorlabs/ DMLP505R) was used to separate the signals of SHG (630 nm) and THG (410 nm). The signals of the SHG and THG will be detected by two individual photomultiplier tubes (PMTs) (Hamamatsu/R928 for SHG and Hamamatsu/R4220P for THG) with band-pass filters (BPF) inserted (Semrock FF02-617/73 for SHG and Semrock FF01-417/60). The objective lens was attached to a 3D stepping motor (Sigma Koki/TSDM40-15X) which can move the imaging plane through computer control. Following Ref.^69^, the pulse group delay dispersion (GDD) at the illumination plane after the objective was − 60 fs^2^, corresponding to a pulsewidth of 28 fs. Since it has been reported that the chromatic dispersion value of water with lipid at 1300 nm is zero^70^ and the group velocity dispersion value of water^71^ at 1262 nm as − 47 fs^2^/mm, we thus expect that the GDD value of 300-μm-thick skin tissues at 1262 nm is on the order of − 10 fs^2^n so that its effect on lengthening the 1262 nm pulse duration is negligible.

### Study protocol

Before the glycerol application, we took the bright-field images and HGM images of the skin sample. Then we applied the glycerol by immersing the whole sample into the solution or topically applied by slightly brush the solution on the epidermal side of the skin. After the glycerol application, we took the bright-filed images and HGM images of the skin sample again as post-glycerol. The condition and parameters of both the imaging system were maintained the same during all the measurements. Three different case types were studied in this research (Table 6). We first studied the case of 100% immersion due to its well-understood and promising OC effect. Two tissues from different parts of the skin from different volunteers were included. Lower concentration glycerol was found less effective^8,17,28,33^ and the effect was not well-studied in previous works. We thus compared the OC effect of 50% glycerol with immersion with the case of 100% glycerol. Two tissues from different parts of the skin from different volunteers were included in the second case type. To further study the OC potential of the safe 50% glycerol with topical application, four tissues from different parts of the skin from different volunteers were included. The results of the third case type, 50% glycerol with topical application, will help future in vivo human skin trials. As 100% glycerol might be harmful to human skin which is not helpful for the in vivo human skin trials, we did not use it for topical application.

**Table 6:** Three different case types of the ex vivo optical clearing study.

Case type	Agent	Method	Application time	Subjects
E100-I90	100% Glycerol	Immersion	90 min	2
E50-I90	50% Glycerol + 50% dd ${H}_{2}O$	Immersion	90 min	2
E50-T90	50% Glycerol + 50% dd ${H}_{2}O$	Topical	90 min	4

### Imaging protocol

For HGM imaging, we took *en face* 2D images (235 μm × 235 μm; 512 × 512 pixels) of the skin at different depths with a 1.8 μm vertical step size between images ranging from the stratum corneum to the reticular dermis to form one 3D image stack. The 2D pixel size is 460 nm, which is close to the previously measured lateral resolution in live human skin^72^. The average power of the laser after the objective was 100 mW, same as previous in vivo clinical trials^42–48,54^. With a laser repetition rate of 105 MHz, the corresponding pulse energy was 0.95 nJ per pulse, with a maximum pulse fluence of 0.18 J/cm^2^ at the focal spot and a corresponding scanning power density of 181 W/cm^2^ at the sample plane. Under the same experimental conditions, pervious clinical studies indicated that the nonlinear photodamage for 1260-nm is far below the threshold to cause the DNA damage and can be negligible^42–48,54^. After HGM imaging of the same imaging condition, previous in vivo clinical studies reported that there was no erythema, pigmentation, crust, or vesicular formation, nor does the volunteer feels heat or discomfort^42–48,54^. For each sample, at least six image stacks acquired at different points of the skin were collected. The number depended on the size of each sample. With a system frame rate of 30 frame/s, each *en face* 2D image were averaged with 15 frames. The THG modality can provide information about the morphological structure of the epidermis^54,73^, but the signal is relatively weak to image the collagen in the dermis. On the contrary, the SHG modality can provide the information of collagen fibers^54^ in the dermis. It is noted that for fair comparison, we have fixed the PMT voltages to be the same throughout the comparison study. In order to get a reasonable image quality in deep layers, the pixel intensity in the uppermost layer (SC) might thus be saturated, which would affect the intensity comparison analysis of SC but not the structure analysis on the layer depth. Furthermore, the image intensity was strongly affected by the imaging system alignment. To ensure a fair comparison, instead of normalization, all the presented pre- and post-glycerol images were acquired by the same system setting which is under a tight performance control, while the operating conditions were constantly checked and confirmed to be the same before and after the glycerol application by taking the images of a calibration sample (gallium nitrite grown on top of double-side polished sapphire substrate), which can generate strong and stable SHG signal on the thin gallium nitrite layer^74^.

### Analysis procedure

The group information will be blinded before the analysis. The person (JHL) who analyzed the HGM image didn’t know whether the stack was from before or post-glycerol. We analyzed two different types of parameters from the HGM skin images to study the effects of the optical clearing, which are the thickness of the skin layers (structure analysis) and the harmonic generation intensities (intensity analysis). The images of THG and SHG modalities were presented with red and green false colors. The images were processed and analyzed using Image J (National Institutes of Health, https://imagej.nih.gov/ij/).

**Figure 11:**
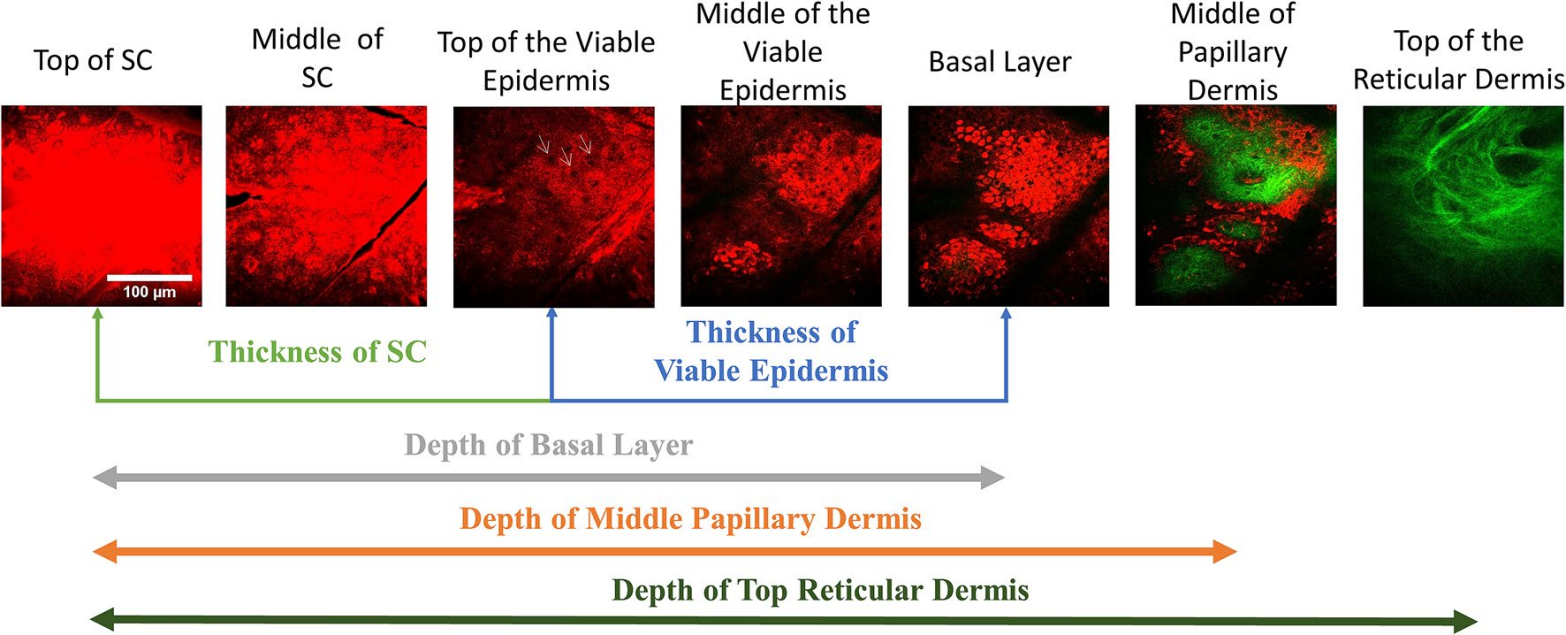
The HGM images of human skin obtained at different depths showing different skin structures. THG and SHG are shown with false red and green colors. The definitions of 5 different parameters related to the layer thickness are displayed. *SC* Stratum corneum. Scale bar: 100 μm. White arrow: cells.

Structure analysis: Thickness variation of the skin tissue after the introduction of OCAs was proved as one of the most important mechanisms in previous studies. Shrinkage or swelling of tissues will affect the scattering coefficient^32^. After the glycerol application, glycerol solution might diffuse into the skin and cause dehydration so as to make the skin shrink. However, the variation of thickness might be different in different layers of the skin (skin structure for the top to the bottom: the stratum corneum, viable epidermis, papillary dermis, and reticular dermis^75^) and might result in different optical clearing efficacies. Five different parameters related to the layer thickness were investigated, which are the thickness of SC, the thickness of viable epidermis, depth of the basal layer, depth of the middle papillary dermis layer, and depth of the top reticular dermis layer^48,69^. On the top of the SC, the strongest THG signal will be generated. We defined the top layer of the SC as where the THG signal started to appear. The thickness of the SC is counted from the top layer of SC to the top layer of the viable epidermis. The top of the viable epidermis (or the bottom of SC) is defined as the number of the existing dark nuclei of the skin epidermis image is more than six. The thickness of the viable epidermis is counted from the top layer of the viable epidermis to the stratum basale layer, which shows strong THG signals of the cytoplasm due to melanin^76,77^. In the area of papillary dermis, the collagen fibers start to appear and show the strong SHG signal^54^. Beneath the papillary dermis, the much thicker reticular dermis is composed of dense collagen fibers. We choose the first skin layer, beneath the papillary dermis, that completely lacks the signal of basale lamina as the top of the reticular dermis. The middle papillary dermis layer is defined as the central layer between the stratum basale layer and the top reticular layer (Fig. 11).

Intensity analysis: The intensity analysis in this study is quite different from the analysis that frequently used in some previous studies. Although provided with a stack of images at different skin depths, we are not going to compare the intensity at the same skin depth before and after the glycerol application by plotting the depth-dependent intensity diagram. After the application of glycerol, layer depth will follow the variation of structures (for example, shrinkage), and it is unsuitable to compare the image intensity at the same depth since the image intensity will be affected by the shift of the layer depth. Based on the high 3D resolution and label-free characteristic of the HGM that enables us to distinguish different skin sub-layers, we implemented the position correction to achieve the image intensity comparison of the same skin structures before and after the glycerol application. We recorded and compared the average THG intensities of five different skin layers before and post-glycerol applications, which include the bottom of stratum corneum, the middle layer of the viable epidermis, the basal layer, the middle layer of the papillary dermis, and the top layer of the reticular dermis. The average SHG intensities of three different depths were recorded, which were the middle layer of papillary dermis, the top reticular dermis, and the layer with the maximum SHG intensity.

The result of the skin thickness and the HGM image intensity was expressed as mean ± standard deviation. Independent sample t-test was used for comparisons. Statistics were performed with the excel software (Microsoft office 2013), and P < 0.05 was considered statistically significant.
